# Assessment of *Acacia Koa* Forest Health across Environmental Gradients in Hawai‘i Using Fine Resolution Remote Sensing and GIS

**DOI:** 10.3390/s110605677

**Published:** 2011-05-27

**Authors:** Rodolfo Martinez Morales, Travis Idol, James B. Friday

**Affiliations:** 1 Charles Darwin Foundation, Puerto Ayora, Galapagos, Ecuador; 2 Department of Natural Resources and Environmental Management, University of Hawai‘i at Mānoa, Honolulu, HI 96822, USA

**Keywords:** forest health, dieback, *Acacia koa*, Hawaii, GeoEye, remote sensing, GIS

## Abstract

Koa (*Acacia koa*) forests are found across broad environmental gradients in the Hawai‘ian Islands. Previous studies have identified koa forest health problems and dieback at the plot level, but landscape level patterns remain unstudied. The availability of high-resolution satellite images from the new GeoEye1 satellite offers the opportunity to conduct landscape-level assessments of forest health. The goal of this study was to develop integrated remote sensing and geographic information systems (GIS) methodologies to characterize the health of koa forests and model the spatial distribution and variability of koa forest dieback patterns across an elevation range of 600–1,000 m asl in the island of Kaua‘i, which correspond to gradients of temperature and rainfall ranging from 17–20 °C mean annual temperature and 750–1,500 mm mean annual precipitation. GeoEye1 satellite imagery of koa stands was analyzed using supervised classification techniques based on the analysis of 0.5-m pixel multispectral bands. There was clear differentiation of native koa forest from areas dominated by introduced tree species and differentiation of healthy koa stands from those exhibiting dieback symptoms. The area ratio of healthy koa to koa dieback corresponded linearly to changes in temperature across the environmental gradient, with koa dieback at higher relative abundance in warmer areas. A landscape-scale map of healthy koa forest and dieback distribution demonstrated both the general trend with elevation and the small-scale heterogeneity that exists within particular elevations. The application of these classification techniques with fine spatial resolution imagery can improve the accuracy of koa forest inventory and mapping across the islands of Hawai‘i. Such findings should also improve ecological restoration, conservation and silviculture of this important native tree species.

## 1. Introduction

*Acacia koa* Gray (koa) is a large evergreen forest tree in the Fabaceae family. For established trees the true leaf is replaced a phyllode, which is the expanded rachis of the true leaf. These are thought to aid in drought tolerance [1]. Koa is an important native tree species in Hawai‘i due to its high economic, ecological and cultural values. Koa serves as the preferred and critical habitat for native insects [1] and threatened and endangered bird species [2], improves soil nitrogen content [3], and creates favorable understory conditions for native plant regeneration [4]. Because of its recognized fine wood quality and conservation values, there is growing interest in establishing koa plantations and forests in former agricultural and grazing lands. Koa exists on most of the main Hawaiian Islands in remaining native forest areas and regenerating second-growth stands across a wide range of elevation (600–2,300 m asl), mean annual precipitation (850–5,000 mm), and soil types [5]. Several studies have found that these environmental gradients have direct effects on various aspects of productivity and ecosystem function. For example, koa productivity generally increases with precipitation, but nutrient availability becomes more limiting due to increased leaching and plant demand [6–8].

Through ground-based inventories of forest cover and health status, investigators have detected instances of koa dieback throughout the ecological range of koa forests on several of the main Hawai‘ian Islands [9,10]. Whether the dieback represents a new disease or a naturally occurring phenomenon is unknown, but field observations indicate that it is becoming increasingly frequent. One pathogen identified in many areas of dieback is the soil-borne fungus, *Fusarium oxysporum* f. sp. *koae*. Upon infection, the fungus blocks the vascular tissue and inhibits movement of resources between shoots and roots. Typical symptoms include yellowing of the phyllodes with subsequent crown thinning until complete defoliation and tree death [9]. Young trees typically die rapidly after the first symptoms appear, but older trees often survive because they successfully isolate the infected tissue and continue to produce new growth from uninfected parts of the tree [10]. In addition, insects such as the koa psyllid (*Psylla uncatoides*) and the coffee twig borer (*Xylosandrus compactus*) are generally not lethal but can impair koa forest health through partial defoliation and cause serious economic damage by altering the stem form [11].

Field studies within diseased tree patches have improved our understanding of the role of pathogens through characterizations of crown symptoms and physiological condition of diseased trees, stand structure and soil conditions. Findings have been limited to plot-level assessments, mainly in accessible areas. There is a need to study the spatial distribution and spread of dieback across broad geographic regions in order to focus restoration and monitoring strategies for early and efficient control and management. Significant koa forest mortality would not only reduce critical habitat for native flora and fauna and allow for invasion by non-native species but would also cause severe economic damage to the timber industry. Because very large plots are often required to quantify large-scale impacts of forest mortality, additional research needs to be done to achieve a more complete assessment of koa forest dieback across the environmental gradients in Hawai‘i, especially in inaccessible areas. Forest pests and diseases commonly disperse at large scales depending on the distribution of the host plant and the presence of suitable environmental conditions for pathogen reproduction and spread. Although it is generally believed that koa dieback distribution is associated with climatic factors, findings have not been reported in the literature. Therefore, in the study of koa dieback, it is crucial to understand the interactions between koa forests and their physical and biological environment.

The analysis of fine spatial resolution multispectral and panchromatic satellite imagery has provided a way to study large areas by allowing visualization of entire landscapes and regions and identification of individual tree species. Due to high temporal frequency of flights over the same area (3 to 4 days), fine resolution satellites such as IKONOS (4 m MS (multispectral), 1 m Pan (panchromatic)) and Quickbird (2.4 m MS, 0.5 m Pan) have facilitated assessments of forest structure, condition and health across multiple spatial and temporal scales [12]. Imagery from these satellites has improved the identification and mapping of individual forest species across entire landscapes. The high spatial resolution allows for delineation of single tree crowns. The multispectral bands allow for determination of variations of canopy greenness within forest stands [13]. This technology is being increasingly applied at the landscape level to quantify and model larger spatial patterns of forest dieback [14]. In particular, the IKONOS satellite has been successfully applied for forest inventory in tropical environments. It has allowed for the mapping of tree crown sizes [15], tree density, species identification, and assessment of temporal changes in individual tree growth and mortality [16]. Other studies have used these data to calculate texture parameters and several vegetation indices as a measure of vegetation greenness and related those to biophysical indices of forest productivity such as tree height, basal area and leaf area index for spatial prediction at the landscape scale [17]. Therefore, similar applications of this technology could facilitate spatial and temporal assessments of koa forest health through detection of forest structure and greenness changes due to forest dieback and to accurately predict its potential distribution at the stand and landscape scales in the islands of Hawai‘i.

The purpose of this study was to apply remote sensing and geographic information systems (GIS) methodologies to characterize the health of koa forests and the spatial distribution of koa forest dieback across an elevation gradient on the island of Kaua‘i. Affected trees largely comprise the forest overstory, which is viewed by satellites. In most cases, as unhealthy trees die, the entire crown gradually changes from healthy green to brown before complete defoliation. We hypothesized that the analysis of high spatial resolution imagery from the new Geoeye1 satellite (2 m MS, 0.5 m Pan) would allow for delineation of not only single-tree crowns but also detection of trees experiencing dieback phases, including complete defoliation on mature trees with dense branching. We also hypothesized that koa forest health and dieback dynamics are largely influenced by changes in environmental conditions such as temperature, rainfall, and relative humidity across the elevation gradient. Therefore, a more thorough and meaningful analysis was carried out through the integration of climatic and remote sensing data using GIS tools in order to illustrate complex environmental interactions that influence koa forest health across environmental gradients in Hawai‘i. A better understanding of the relationship of koa dieback to environmental factors will help foresters and land managers select better sites for restoration of koa forests.

## 2. Materials and Methods

### 2.1. Site Description

**Figure 1.**
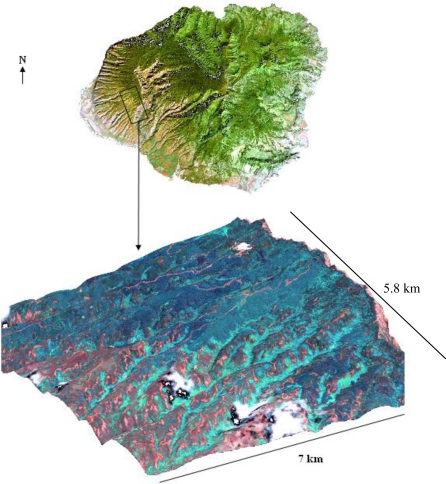
Landsat satellite image of the Island of Kaua‘i (top) and GeoEye1 satellite image (bottom) overlaid on a digital elevation model 3D surface view depicting the elevation gradient.

Areas of koa dieback have been increasingly observed in the Pu‘u Ka Pele and Nā Pali Kona Forest Reserves on the Island of Kaua‘i (Figure 1). The study area included koa stands growing on highly weathered soils along an elevational gradient of 600–1,200 m asl. Mean annual temperature declines with elevation from 20–17 °C. Mean annual precipitation increases with elevation from 750 to 1,500 mm [18]. Monthly precipitation increases with elevation from about 60–90 mm in the dry summer months and 100–200 mm in the wet winter months. Koa remains evergreen despite seasonal variations in rainfall. Koa stands in this area are highly mixed with several introduced tree species including *Eucalyptus* spp. (eucalyptus), *Pinus elliottii* Engelm. (slash pine) and *Grevillea robusta* A. Cunningham ex R. Br. (silk-oak).

### 2.2. Image Analysis and Classification

Land cover types were classified into healthy koa stands, unhealthy koa stands (those exhibiting dieback symptoms), other tree species, and other land cover types. This classification followed a progression from data collection in the field for image training, processing of raw data from satellite images, exploratory analysis of spectral properties for each designated cover class and final classification based on differentiation of the unique reflectance characteristics of each class across the MS bands using supervised methods.

#### 2.2.1. Satellite Imagery

**Figure 2.**
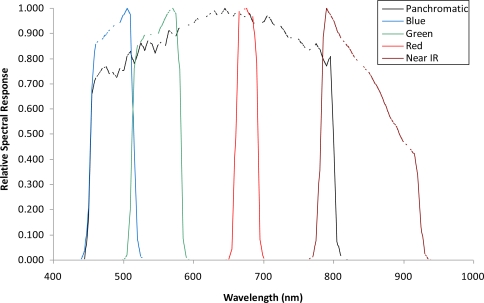
GeoEye1 satellite relative spectral response in the visible and NIR spectral regions.

A set of cloud-free images with a near-nadir view from the GeoEye1 satellite (GeoEye, Inc., Dulles, VA, USA) covering potential areas of koa forest dieback across the environmental gradient was obtained on July 2009 (Figure 1). The images consist of 2.0-m pixel MS bands in the visible spectrum including the blue (450–510 nm), green (510–580 nm), red (655–690 nm) and near-infrared (NIR) (780–920 nm), and a 0.5-m pixel panchromatic band that includes the visible and NIR spectral regions (450–829 nm) (Figure 2). All bands were orthorectified to a horizontal accuracy of less than 5 m using a 10-m-pixel resolution digital elevation model (DEM) obtained from the Hawai‘i Statewide GIS and the nearest-neighbor resampling method using rational polynomial coefficients in ENVI (ITT Visual Information Solutions, Boulder, CO, USA). The orthorectified images allowed for more accurate location of targeted trees and did not show geometric artifacts that are typically found in images over terrain with steep topographic changes. Therefore, the quality of the remote sensing data used for this study ensures the highest pixel resolution available with highly accurate geometric and topographic corrections important in sloped areas. Since the spectral range of the single Pan image and the four MS bands is similar, the MS bands were fused with the Pan band to produce four MS pan-sharpened bands (MSPan) at 0.5 m pixel resolution using the spectral-sharpening technique of [19,20], who demonstrated that a pan-sharpened multispectral image maintained the radiometric accuracy of the original bands.

#### 2.2.2. Selection of Training Sites

Previous to the image analysis process, an initial field survey was conducted during October 2008 for landscape recognition and collection of ground-truth information that allowed for identification of land cover classes existing across the entire area (tree and shrub species and grasses). The survey included the collaboration of forest health experts and managers from the Hawai‘i State Division of Forestry and Wildlife (DOFAW) for the identification of koa trees and stands experiencing dieback. This procedure was also necessary to determine and select training sites (groups of pixels representing a class) at known locations. Ten dying and/or dead koa stands were identified and geolocated across the environmental gradient using a Trimble GeoXT GPS with sub-meter accuracy. Koa trees were selected on the basis of showing symptoms of leaf chlorosis, defoliation, and death without any determination being made of the cause of dieback, whether biotic or abiotic. Ideal koa dieback sites were surrounded by healthy koa stands to ensure that the identified dying or dead trees were koa. Additional sites that represented 100% coverage of healthy koa and other abundant species in the area such as eucalyptus, pine, silk-oak, and other cover classes such as grass-soil mixture, bare soil, asphalted roads and water bodies were also geo-located. Geo-located points were overlaid on a natural color image composite in order to delineate training sites by drawing polygons around the GPS mark. A true color composite allowed more accurate visual inspections to clearly define training site boundaries. Ten polygons that included about 150 contiguous pixels (∼37 m^2^) were drawn per cover class across the entire image area. The total number of training pixel values (digital numbers, DNs) per cover class (about 1,500) were extracted per band and used for exploratory analysis to determine the spectral separation prior to image classification. The Shapiro-Wilk method was used to test how well a Normal distribution fit the DNs of the four bands across all training sites. Homogeneity of variance of all DNs among classes was determined with O’Brien’s test [21]. Both tests were carried out using JMP software (SAS Institute Inc., Cary, NC, USA).

#### 2.2.3. Spectral Analysis

An exploratory analysis was carried out to determine which MS and/or MSPan bands could maximize separation between training classes (koa dieback, healthy koa, pine, eucalyptus, silk-oak and other land cover types). Due to the high correlation between contiguous MS bands, a feature selection procedure prior to image classification was done using analysis of divergence among classes. This allowed for selection of important bands that maximized differentiation of classes and optimized classification by reducing the amount of redundant information. Two feature selection procedures that included all training classes and spectral bands were tested. The first was the Jeffreys-Matusita (J-M) distance analysis, which provides a listing report of the divergence values for every possible pair of classes for the bands being studied. A calculated divergence close or equal to two indicates maximum separation, and a zero value means that the classes are inseparable [22]. These numbers were compared to determine which set of bands was the most useful for classification. The second procedure included the average of the total DNs values for each training class per band in order to determine and graphically illustrate their pseudo-spectral signatures. This procedure allowed a better depiction and comparison of the DNs range unique to each class and the amount of overlap across the spectral bands.

#### 2.2.4. Image Classification

The Maximum Likelihood Classifier (MLC) included in ENVI was used for detailed classification of koa stands by health status across the elevation gradient. The classifiers assign every individual pixel in the image to a training class based on spectral pattern similarities across the bands using Bayesian probability theory [23]. The algorithm estimates a probability distribution which describes the chance of a given pixel value being a member of a particular class. Pixels are assigned on the basis of the shortest probabilistic distance from class means, depending on the shape, size and orientation of training samples. MLC results in fewer classification errors if the assumption of a normal distribution for each training class is met across all spectral bands [24,25]. To validate the image classification, a second field survey was made on December 2009 for the identification and geo-location of a set of ten testing sites that included a mixture of healthy koa stands, unhealthy koa stands and the remaining cover classes. The procedure used to define and extract training DNs was applied to obtain testing pixel values for each class from the classified image. Thus, a testing site included about 150 pixels per class. Across the 10 testing sites, this resulted in 1,500 testing pixels per class. A confusion matrix was then calculated to determine the classification accuracy. The matrix determined the percentage of pixels that were classified into each class.

### 2.3. GIS Analysis

#### 2.3.1. Climate Data

Climatic maps of mean monthly temperature (°C), precipitation (mm) and maximum and minimum relative humidity (%) were obtained in raster format at a resolution of 200 m per pixel from *The Rainfall Atlas of Hawaii* [18]. Using the temperature data, the monthly potential evapotranspiration (mm) (PET) was calculated with the algorithm of Thornthwaite (1944) (Formula 1), which defines potential evaporation as “the water loss which will occur if at no time there is a deficiency of water in the soil for use of vegetation”.

(1) $$\text{PET}(\text{mm}/\text{month})=\text{dl}\times (\text{dm}÷360)\times 16\times {\left(10\times \text{T}÷\text{I}\right)}^{\text{a}}$$

where:

dl: daylength in hours
dm: days in a month
T: mean monthly temperature (°C)
I: monthly heat index given by (T ÷5)^1.5^
a: 0.49 + 0.0179 × (I − 7.71^−5^)×(I^2^ + 6.75^−7^) × I^3^

Maps of mean annual temperature (MAT) and precipitation (MAP) were also obtained at a resolution of 200 m per pixel [18]. Therefore, PET was calculated both annually and monthly. The difference between monthly PET and MAP was calculated to determine the number of dry months over the year. The annual PET/MAP ratio was calculated since it is used as an indicator of vegetation health and productivity to classify vegetation world life zones [26] and has been applied to Hawai‘i’s diverse climatic and vegetation zones [27].

#### 2.3.2. Climatic Zones

In order to estimate percent of the koa forest affected by dieback and to compare it with the environmental factors, the study area was divided in environmental zones across the gradient using MAT and MAP values. Maximum and minimum relative humidity were similar across the study area (97% and 68%, respectively). The various levels of rainfall and temperature were combined to create climatic zones. Pixels from each layer were reclassified to unique numbers representing three levels of MAP: low (750–1,000 mm, P_low_), medium (1,000–1,250 mm, P_mid_) and high (1,250–1,500 mm, P_high_) and three levels of MAT: low (17–18 °C, T_low_), medium (18–19 °C, T_mid_), and high (19–20 °C, T_high_) for a total of nine classes. Reclassified layers were added to obtain nine climatic zones ordered by levels of increasing temperature (T_low_ + P_low to high_, T_mid_ + Plow to high and T_high_ + P_low to high_) using ArcGIS (ESRI, Redlands, CA, USA). Because the climatic zones represented relatively broad areas, three sub-zones encompassing a similar number of pixels were delineated within each climatic zone resulting in a total of 27 sub-zones. All pixel values of MAP, MAT, PET and PET/MAP were extracted per sub-zone for calculation of averages.

### 2.4. Environmental Effects Assessment

In order to investigate important environmental controls over koa health and dieback, the classified image was overlaid on the climatic zones map to calculate the total area of pixels classified as healthy koa and koa dieback per sub-zone. Since climatic zones varied in size, the ratio of healthy to unhealthy koa cover (healthy koa:unhealthy koa ratio) within sub-zones was calculated to allow an equivalent comparison of the distribution of koa dieback across climatic zones in the elevation gradient. Smaller ratio values indicate a higher prevalence of koa forest dieback. To quantify the relationship of environmental variables to the magnitude of the ratio, the average values of MAP, MAT, PET and PET/MAP were extracted from each sub-zone and analyzed by two statistical procedures using JMP software. The first included the identification of individual environmental variables (MAP, MAT, PET and PET/MAP) that could be related to the healthy koa:unhealthy koa ratio through linear regression and analysis of R^2^ and error distribution to determine the strength of the relationship. The second procedure included the selection of best prediction models of the ratio from all possible combinations of MAP, MAT, PET and PET/MAP using forward stepwise regression. Two model-selection parameters were used to determine a parsimonious number of environmental variables. The adjusted R^2^ from linear regression provides a proportion of variability in a data set that is accounted for by the statistical model, adjusted according to the number of environmental variables. It provides a measure of how well future healthy koa:unhealthy koa ratio estimations are likely to be predicted by the model. Therefore, a higher R^2^ indicates more accurate model predictions. The Akaike Information Criterion (AIC), based on maximum likelihood to minimize the error sum of squares with the minimum number of parameters, was used to select the optimal number of parameters to include in a model. The model that has the smallest AIC value is considered optimal.

## 3. Results

While the J-M distance feature selection procedure from the analysis of MS bands resulted in values <1.5 for the class pair unhealthy koa and grass-soil mixture, the analysis of MSPan bands resulted in values from 1.99 to 2 for most pairs of cover classes across the gradient. Therefore, this analysis indicated that the MS bands did not allow accurate classification of koa dieback due to large overlap with grasses, but that all MSPan bands were important to differentiate cover classes. Individual koa crowns were generally less than 10 m^2^, so only the MSPan imagery provided enough detail to characterize them. Therefore, only MSPan were further analyzed to determine class pseudo-spectral signatures.

**Figure 3.**
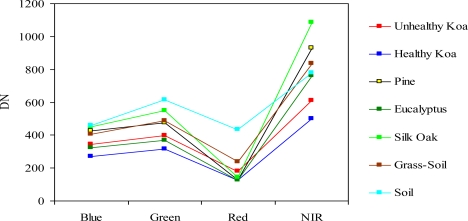
Pseudo-spectral signatures of tree species and land cover classes across the visible and NIR spectral bands in digital numbers (DN).

There was a clear spectral separation between healthy and unhealthy koa, as the latter had higher reflectance values across the four bands (Figure 3). Koa stands experiencing dieback had also consistently lower reflectance values than the grass-soil mixture class, indicating that the four bands contained important information to accurately differentiate them. The NIR followed by the green and blue bands provided the best spectral separation of healthy koa forests from all other classes, as the koa pseudo-spectral signature was the lowest in these bands. The red band contained the lowest spectral values for all vegetation classes and a large degree of overlap, especially among tree species. Although this band separated healthy from unhealthy koa stands and the grass-soil mixture, a large spectral overlap was observed between healthy koa with the other major tree species (Figure 3). Therefore, the red band was discarded from the classification process using MLC, since an accurate classification of healthy koa stands was also needed.

**Figure 4.**
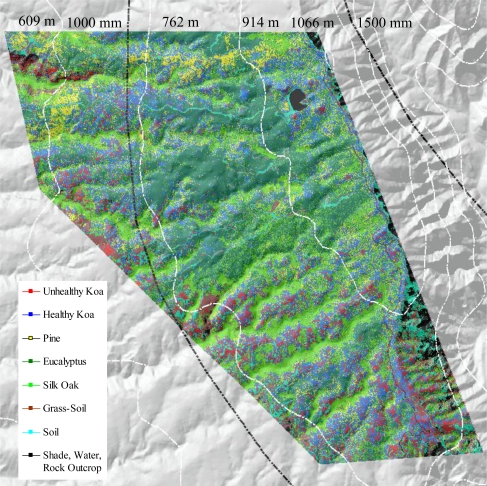
Image classification of the entire elevation gradient overlaid on a digital elevation model 3D surface view. Black lines represent isohyets in millimeters of rainfall and white lines are elevation contours in meters.

**Figure 5.**
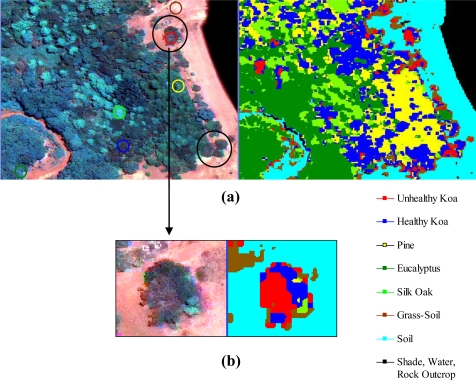
Close up of a natural color composite (left) showing the location of geo-located testing sites (colored circles) for comparison with resulting classes in the classified image (right). Circle colors match class colors in the classified image. Black circles in the color composite represent areas containing a mixture of unhealthy (brown) and healthy koa (green) stands.

The use of MSPan bands and the analysis of their relative contributions to optimally differentiate healthy and unhealthy koa allowed the classification of these two classes from other vegetation classes across the environmental gradient (Figure 4). The visual comparison of the natural color composite of an area that was included in the analysis of testing sites with the classified image illustrated separation of healthy koa forests from pine, eucalyptus and silk-oak even if they were highly mixed (Figure 5(a)). The differentiation of koa stands exhibiting dieback symptoms from contiguous healthy koa stands and grass-soil mixture was also clear and consistent with the position of validation sites (Figure 5(b)). The confusion matrix indicated that MLC accurately differentiated healthy from unhealthy koa stands (Table 1). While most pixels representing unhealthy koa in the testing site were accurately classified (98.6%), a small percentage of pixels representing other classes were misclassified as unhealthy koa (<0.03% for tree species and 2.30% for shadow-asphalt). The majority of pixels representing healthy koa in the testing sites were accurately classified (87.10%), but 11.30% and 1.50% of those pixels were misclassified as pine and eucalyptus, respectively. Only 0.77% and 3.80% of pixels representing eucalyptus and pine were misclassified as healthy koa. Pixels representing pine, eucalyptus and silk-oak were in general classified with high accuracy (88.5%, 94.3% and 99.9%, respectively).

**Figure 6.**
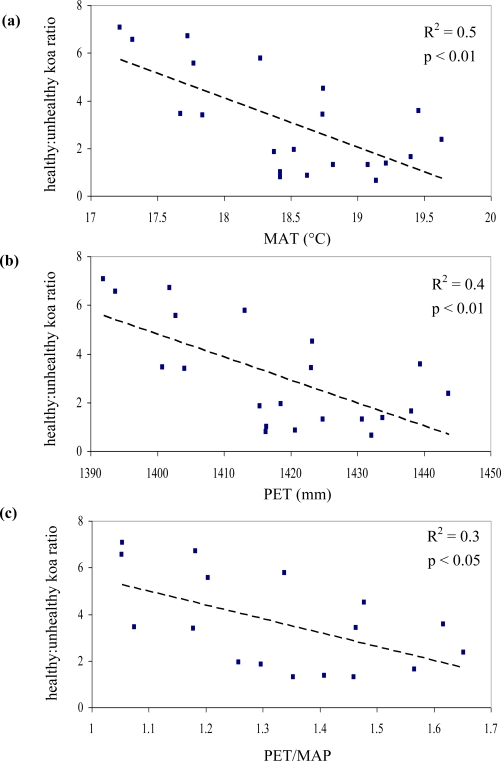
Relationship between healthy:unhealthy koa ratio and climatic zones of increasing temperature **(a)** MAT, **(b)** PET and **(c)** PET/MAP.

Out of the nine possible climatic zones only P_low_T_low_ did not exist in the study area. Of the other eight climatic zones, one (P_high_T_high_) did not contain pixels representing healthy or unhealthy koa stands. Therefore only 7 zones (21 sub-zones) were included in the analysis (Table 2). The healthy:unhealthy koa ratio gradually decreased as MAT increased (R^2^ = 0.5, p < 0.01, Figure 6(a)), indicating that koa dieback was greater at warmer temperatures. The ratio also gradually decreased with increasing PET (R^2^ = 0.4, p < 0.01, Figure 6(b)). The ratio of potential evapotranspiration to precipitation (PET/MAP), calculated as an index of drought stress, was significantly correlated to koa dieback (R^2^ = 0.3, p < 0.05, Figure 6(c)), but not as strongly as PET alone. The overall distribution of the healthy:unhealthy koa ratio was highly scattered when related to climatic zones ordered by increasing MAP (R^2^ = 0.03, p > 0.05) and the number of dry months over the year (R^2^ = 0.02, p > 0.05), indicating that rainfall by itself was not a strong controlling factor for koa forest health. The model selection procedure indicated that the inclusion of MAT, MAP and PET resulted in the optimal prediction of the healthy:unhealthy koa ratio (Table 3), even though the model including MAP alone was not significant. Inclusion of the PET/MAP ratio did little to improve the models.

## 4. Discussion

The use of integrated MSPan bands at 0.5-m pixel resolution and the analysis of their relative contributions to differentiate healthy from unhealthy koa stands across the climate gradient was an effective methodology for large-scale characterization and mapping of koa forest health. The high spectral variability characteristic of trees experiencing dieback due to changes from green to yellow to brown, small crown sizes and visible gaps between tree canopies, were captured at this pixel resolution and were differentiated from similar reflecting objects such as green-dried grass mixtures and soil surfaces covered with plant residues. The original MS bands are captured at a 2-m pixel resolution by the GeoEye-1 satellite, which was too coarse for effective detection and delineation of koa crowns experiencing dieback, especially fully defoliated trees. While the NIR band contained the highest reflectance values for all vegetation classes and varied the most between healthy and unhealthy koa and among tree species, the red band contained minimum reflectance and varied the least among vegetation classes. This is in agreement with the typical green vegetation spectral pattern of low reflectance in the red region due to chlorophyll absorption and strong reflectance in the NIR associated with internal leaf structure [28,29]. Healthy trees with greener canopies should utilize more short-wave radiation and reflect more near-infrared radiation. The leaf structure of the major tree species in this area (a phyllodinous *Acacia*, a coniferous pine, and two broadleaved species in different taxonomic orders) differed sufficiently to allow for distinction based on reflectance in the NIR region.

The image classification procedure provided additional tools to increase the accuracy of koa forest health characterization across the environmental gradient. As indicated by testing sites, the image classification using MLC was highly accurate, even when forest stands had mixed species, confirming that their unique spectral characteristics could be captured by the satellite sensors. Eleven percent of the area representing pine was misclassified as healthy koa, indicating the need to increase the number of training sites in future classifications. The Shapiro-Wilk normality test revealed that none of the bands deviated from a normal distribution across the training sites (p > 0.01), so the MLC classification results using the blue, green and NIR bands should be reliable. When normality of training classes is met across the spectral bands, other studies have also obtained optimal classification results using MLC [24,25].

Temperature, precipitation, and potential evapotranspiration were highly correlated along the elevational gradient. The area of healthy:unhealthy koa corresponded linearly to changes in temperature and PET, with koa dieback at higher relative abundance in warmer areas and higher PET. This is in agreement with casual ground-level observations of the prevalence of koa dieback, which seems to be increasing at the site. Experimental plantings of koa below ∼600 m often result in very high mortality (80–90%) by 10 years of age [30]. There was no significant correlation of koa dieback with precipitation or the use of PET/MAP as an index of drought stress. Past studies have found strong positive relationships of koa indices of productivity with MAP [7,8]. The one study along this gradient found that koa basal area, but not woody biomass increment or leaf area, increased as precipitation and elevation increased [8]. More recent work found that indices of productivity increased with MAT along a gradient where precipitation was relatively constant [25]. However, warmer climatic conditions also may increase the prevalence and virulence of plant pathogens, especially if the host species has low resistance. While wetter conditions generally also favor fungal growth, along this gradient the wetter areas were also cooler. At this location, it seems that temperature rather than moisture affects the degree of koa forest dieback. Areas of koa dieback can and should be checked for the presence of *F. oxysporum* to determine whether the koa wilt disease or other pests or pathogens or abiotic stressors are the cause of dieback.

The characterization and distribution assessment of healthy and unhealthy koa forests across the environmental gradient developed in this study using remote sensing and GIS analysis can support mapping of koa health and dieback at various spatial scales, depending upon the expected distribution of koa stands. The methodology developed in this project was highly practical, as it required minimal image preprocessing and simple exploratory analysis and classification algorithms. The field work required to develop training and testing sites was straight-forward and took less than 5 days to complete. Comprehensive ground surveys or collection and analysis of aerial imagery would undoubtedly take much longer and cost much more. This approach can also be applied to monitoring the health of other common native species, such as ‘ōhi‘a lehua (*Metrosideros polymorpha*). More generally, the ability to differentiate tree species suggests this approach can be used for more general forest cover mapping. Highly diverse stands may pose a challenge in terms of developing sufficient training and testing sites for all possible classes, but the resolution of the satellite images is sufficient to detect individual crowns of mature trees. This capability is particularly useful for forest managers and government agencies directly in charge of forest restoration and conservation programs.

## 5. Conclusions

The spectral analysis and classification of GeoEye1 satellite imagery proved to be a useful methodology for the characterization of koa forest dieback across a 500-m elevation (3 °C MAT) gradient on the island of Kaua‘i. In spite of the high reflectance variability associated with koa tree crowns experiencing dieback, the high spatial resolution and the multispectral bands allowed for separation of healthy from unhealthy koa stands along the gradient. Koa dieback was positively correlated with temperature (MAT) and potential evapotranspiration (PET) but was not correlated with precipitation (MAP) nor with the ratio of potential evapotranspiration to precipitation (PET/MAP). The multispectral bands also helped characterize unique reflectance properties of healthy koa forests as a whole, which allowed for clear differentiation from other tree species in the landscape. Therefore, the use of fine spatial resolution satellite imagery can improve not only assessments of koa forest health but also the accuracy of vegetation cover mapping. Results from this project reinforce local government efforts to characterize koa forest dieback distribution across the islands of Hawai‘i in order to develop management strategies that could improve koa forest restoration and sustainable development of koa silviculture.

## Figures and Tables

**Figure 7.**
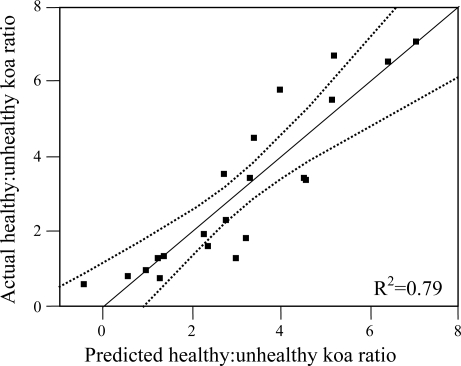
Actual *vs.* predicted healthy:unhealthy koa ratios using the best model selected from the stepwise regression analysis that included MAT, MAP and PET. Dotted lines represent 95% confidence limits.

**Table 1.** Assessment of class overlap among cover classes. Numbers in bold represent the particular class to which the greatest percentage of pixels in the testing site were classified. Columns add up to 100%.

Class	Unhealthy Koa	Healthy Koa	Pine	Eucalyptus	Silk-Oak	Soil	Grass-Soil	Shadow-Asphalt
Unhealthy Koa	**98.6**	0	0.03	0.02	0	0.03	0	2.3
Healthy Koa	0	**87.1**	3.8	0.77	0	0	0	0.04
Pine	0	11.3	**88.5**	1.36	0	0	0	0
Eucalyptus	0	1.5	7.5	**94.3**	0.03	0	0	0
Silk-Oak	0	0.04	0.14	3.16	**99.97**	0	0	0
Soil	0	0.06	0	0.04	0	**94.5**	0	1.99
Grass-Soil	1.4	0	0	0	0	5.44	**100**	1.37
Shadow, Asphalt	0	0	0.03	0.35	0	0.03	0	**94.3**
Total	100	100	100	100	100	100	100	100

**Table 2.** Sub-zone averages of healthy:unhealthy koa ratios (KR) and environmental variables per climatic zone.

ID	Climatic Zones	KR	MAT (°C)	MAP(mm)	PET(mm)	PET/MAP
1	P_mid_T_low_	5.2	17.8	1,183.4	1,403.1	1.2
2	P_high_T_low_	5.7	17.4	1,317.9	1,395.0	1.1
3	P_low_T_mid_	3.0	18.8	971.1	1,423.9	1.5
4	P_mid_T_mid_	3.2	18.4	1,096.8	1,415.9	1.3
5	P_high_T_mid_	0.8	18.5	1,304.3	1,416.9	1.1
6	P_low_T_high_	2.5	19.5	897.0	1,440.5	1.6
7	P_mid_T_high_	1.1	19.2	1,092.7	1,432.3	1.3

**Table 3.** Model selection through stepwise regression for healthy:unhealthy koa ratio (KR) predictions using optimum combinations of environmental factors. k = number of factors, AIC = Akaike Information Criterion.

Model	k	Intercept	R^2^	AIC	p
KR = MAT	1	41.2	0.42	22.3	p < 0.01
KR = PET	1	137.4	0.41	22.9	p < 0.05
KR = PET/MAP	1	5.7	0.02	34.3	p > 0.05
KR = MAP	1	0.57	0.02	34.5	p > 0.05
KR = MAP + MAT	2	88.5	0.71	8.70	p < 0.01
KR = PET + (PET/MAP)	2	271.6	0.72	7.86	p < 0.01
KR = MAP + MAT + (PET/MAP)	3	47.8	0.74	7.30	p < 0.01
KR = MAP + MAT + (MAP x MAT)	3	81.8	0.74	6.90	p < 0.01
KR = MAT + (PET/MAP)	2	68.8	0.74	6.20	p < 0.01
KR = MAP + MAT + PET + (PET/MAP)	4	−1298.4	0.77	5.13	p < 0.01
KR = MAT + PET + (PET/MAP)	3	−937.5	0.78	3.80	p < 0.01
KR = MAP + MAT + PET	3	−1229.3	0.79	3.16	p < 0.01
